# The Body Image Approach Test (BIAT): A Potential Measure of the Behavioral Components of Body Image Disturbance in Anorexia and Bulimia Nervosa?

**DOI:** 10.3389/fpsyg.2020.00030

**Published:** 2020-01-31

**Authors:** Tanja Legenbauer, Anne Kathrin Radix, Eva Naumann, Jens Blechert

**Affiliations:** ^1^LWL University Hospital Hamm for Child and Adolescent Psychiatry, Ruhr-University Bochum, Hamm, Germany; ^2^Department of Clinical Psychology and Psychotherapy, Eberhard Karls University of Tübingen, Tübingen, Germany; ^3^Department of Psychology, Centre for Cognitive Neuroscience, University of Salzburg, Salzburg, Austria

**Keywords:** body image, body image avoidance, body checking, behavior approach test, eating disorder

## Abstract

A disturbed body image with fluctuating behavioral patterns of body related avoidance (BA) and body checking (BC) characterizes individuals with eating disorders (EDs) such as anorexia (AN) or bulimia nervosa (BN). So far, these behavioral body image components are mostly assessed via self-report instruments thereby neglecting their behavioral and partially automatic characteristics. Therefore, behavioral measures of BA and BC are needed. The present study investigates a behavioral assessment task for BA and BC in a sample of patients with diagnosed EDs and healthy controls. The sample consisted of 40 women diagnosed with either BN (*N* = 19) or AN (*N* = 21; ED sample) and 24 non-eating disordered, healthy female controls (HC). Within the Body Image Approach Task (BIAT) participants viewed photos of their own body (self-image) and a matched control body (other-image) by zooming the photos closer toward them (image became more focused) on the screen. The BIAT yields zoom-levels recorded separately for self- relative to other-images. Further measures were attractiveness ratings of these body images as well as questionnaire measures of BA, BC, and general ED symptomatology. Results showed that despite strong body dissatisfaction and clearly negative ratings of self- relative to other-images in both EDs, no group differences were found in approach to self-images on zoom-level as measured with the BIAT. Correlational analysis in each group indicated that zoom-level was positively related to BA scores in the HC group only. Yet, stepwise regression analyses revealed that attractiveness ratings explained most of the variance accounted by BA in predicting zoom-level. In sum, the BIAT seems suitable to assess BA and self-rated body attractiveness, but only in healthy individuals with subclinical levels on these constructs. It does not seem to capture the body image satisfaction or the behavioral components of body image disturbances in AN or BN or it conflates the opposed influences of BA and BC. Further experimentation is needed to adapt measures of behavioral body image components to the processes evoked in patients with ED during confrontation with body images.

## Introduction

Eating disorders (ED) are characterized by pervasive body image disturbances (BID). In Anorexia nervosa (AN), perceptual and attitudinal aspects of BID are classified as necessary diagnostic criteria in the Diagnostic and Statistical Manual for Mental Disorders (DSM-V, American Psychiatric Association [APA], 2013); whereas Bulimia nervosa (BN) criteria require presence of the attitudinal aspects only. This emphasizes the multifaceted nature of BID symptoms which range from perceptual deficits (e.g., seeing oneself fatter than one is), cognitive-affective/attitude distortion (e.g., thinking negatively about one’s body) to dysfunctional body-related behaviors such as checking (BC) and avoidance behavior (BA; e.g., Cash and Deagle, 1997; Legenbauer et al., 2013, 2017; Vossbeck-Elsebusch et al., 2015). Evidence for perceptual and cognitive-affective BID symptoms in ED patients is accumulating, whereas research focusing on body-image related *behavior* is still scarce. This neglected component however seems to exert significant influence on maintenance processes (Fairburn et al., 2003; Shafran et al., 2004; Williamson et al., 2004; Vossbeck-Elsebusch et al., 2015) by for example reducing body-related anxiety through certain safety behaviors (Mountford et al., 2006; Radix et al., 2018).

In order to understand and treat this BID component successfully, valid and comprehensive assessment instruments of BC and BA are required. Up to now, the behavioral component of BID has been assessed via self-report measurements such as the Body Checking Questionnaire (BCQ; Reas et al., 2002) or the Body Image Avoidance Questionnaire (BIAQ; Rosen et al., 1991). Results of studies using these self-report instruments show significant correlations with other aspects of BID and with body weight (e.g., Rosen et al., 1991; Legenbauer et al., 2007; Mountford et al., 2007; Bamford et al., 2014). This former research may be limited as self-report-based assessments can lead to biased results due to self-presentation tendencies and insensitivity to more automatic and implicit behaviors (with possible differences between AN and BN and interferences with severity). To overcome this limitation and to broaden the understanding of behavioral BID in EDs, behavioral assessment should be implemented.

While there is recently a growing number of studies that used variations of the Approach-Avoidance-Task (AAT) in the context of BID or disturbed eating behavior, there are only three studies known to us that tried to capture BA and BC with a behavioral assessment. The first one applied a stimulus response compatibility task in a student sample, where participants were asked to categorize pictures of models either as thin or chubby by moving a manikin symbol to or away from the model picture (Woud et al., 2011). Results showed faster approach than avoidance to thin models; no difference in approach-avoidance tendencies was found for chubby models. The approach – avoidance tendencies were associated to general symptoms of ED (thin internalization, drive to thinness, body dissatisfaction, etc.). More recently, Dondzilo et al. (2019) applied an AAT in undergraduate females with thin-ideal vs. non-thin pictures. Results confirmed hypothesized approach tendencies toward thin-ideal bodies and showed an avoidance bias for non-thin body pictures. Higher levels of body dissatisfaction, thin-ideal internalization and dietary restraint were associated with greater approach bias, whereas avoidance behavior was not correlated with self-reported levels of eating disturbances and BID. There is only one study (Leins et al., 2018) that applied the AAT in women with self-reported eating disturbances (categorized as ED symptoms present vs. absent based on Eating Disorder Examination Questionnaire cut-off scores according to Mond et al., 2004): participants were asked to operate a joystick to either push or pull images of normal weight or underweight pictures toward or away from them (images would zoom larger in proportion to joystick pull movement and zoom smaller for push movements). They failed to replicate the former finding of an approach bias toward thin pictures. Crucially, however, none of these studies used pictures of the participants’ own body, neglecting empirical data that emphasize biased information processing in particular for own body related stimuli (e.g., Jansen et al., 2005, 2006; Roefs et al., 2008; von Wietersheim et al., 2012; Tuschen-Caffier et al., 2015). Thus, further exploration with additional methodology is warranted, in order to enhance the understanding of these behavioral BID components.

Consequently, we designed a task that confronted patients with EDs with a picture of their own body (self-picture) and compared it to a weight matched picture of another woman’s body (other-picture). We focused on picture-based approach behavior as we reasoned this to be a laboratory analog for self-confrontation with regard to mirror exposures. Self-pictures might elicit the experience of negative emotions (e.g., disgust or shame) and thus less of an approach to the pictures, maybe particularly in individuals with BA. A different pattern is predicted under the BC perspective: a precise inspection of the picture (zooming in and increasing focus), potentially motivated by the desire to engage in safety behavior, e.g., looking at certain points of the body to reduce the negative feelings. Given that BID, in particular the behavioral components are best investigated and probably most prominent in the classical EDs such as AN and BN, we recruited a mixed ED sample with patients diagnosed with either AN or BN. This mixed ED group was contrasted against matched healthy controls. However, as findings on differences between AN and BN regarding BC and BA or body dissatisfaction and picture ratings have been inconsistent (Legenbauer et al., 2007, 2017), we also explored differences between these two groups in a second set of analyses. Participants underwent the above described task alongside picture-based attractiveness/satisfaction ratings and established psychometric measures of BA and BC.

We assumed that both AN and BN patients would evaluate their own body as less attractive and report higher dissatisfaction with their own body compared to healthy controls. Consequently, we assumed that AN and BN patients would show stronger avoidance of self-pictures compared to other-pictures. In contrast, healthy controls might inspect their own body more closely (i.e., higher zoom-levels) than other-pictures. In addition, we expected opposing correlational patterns for BA and BC. Finally, we hypothesized that self-reported behavioral BA and BC levels predict zoom-level.

## Materials and Methods

### Participants

The study represented an exploratory extension of a study program on body image in AN and BN sponsored by the German Research Foundation (Tuschen-Caffier and Ansorge; TU 78/6-1.). The present sample consisted of 40 women diagnosed with an eating disorder (ED; 19 with BN, 21 with AN), and 24 healthy female controls (HC). Groups were matched on age and education on the group level. Schizophrenia spectrum disorders, bipolar disorder, substance abuse or dependence and neurological disorders served as exclusion criteria for the ED group, any lifetime mental disorder according to DSM-IV for the HC group. The German version of the Eating Disorder Examination (EDE, Hilbert and Tuschen-Caffier, 2006) and the Structured Clinical Interview for DSM-IV (SCID, Wittchen et al., 1997) were used to diagnose EDs, as well as other psychiatric disorders, respectively. The following comorbid disorders were found in the ED group (BN/AN): major depression (*N* = 8/*N* = 5), dysthymia (*N* = 3/*N* = 1), borderline personality disorder (*N* = 4/*N* = 2), posttraumatic stress disorder (*N* = 4/*N* = 1), social phobia (*N* = 2/*N* = 1), obsessive-compulsive disorder (*N* = 1/*N* = 0), and panic disorder with agoraphobia (*N* = 1/*N* = 0). Five BN patients reported a history of AN.

### Clinical Interviews

#### Eating Disorder Examination (EDE)

The German version of the EDE (Hilbert and Tuschen-Caffier, 2006) was administered. The EDE is a semi-structured interview assessing ED specific symptoms that have occurred within the previous 28 days. Amount and frequency of these characteristics are assessed. Scores can be calculated for four subscales: “eating concern,” “weight concern,” “shape concern,” and “restraint.” It assesses further relevant characteristics of eating disordered behaviors and attitudes with 14 single items. The German version provides good internal consistencies for the subscales (α = 0.73–0.86) and the total score (α = 0.93). Interrater reliability (*r*) for items ranges from 0.80 to 1.00, and for the subscales (*r* = 0.92–0.99).

#### Structural Clinical Interview for DSM-IV TR Axis I Disorders (SCID I)

The SCID I (SCID I; First et al., 1996; German version: Wittchen et al., 1997) is a semi-structured interview for making DSM-IV axis I diagnoses. Its validity has been shown in many studies.

### Self-Report Measures

#### Eating Disorder Examination Questionnaire (EDE-Q)

The EDE-Q (Fairburn and Beglin, 1994) assesses the degree of eating disturbances and BID for the past 28 days across the following four dimensions: “restraint eating,” “eating concerns,” “weight concerns,” and “shape concern.” The German version of the EDE-Q (Hilbert et al., 2007) shows good convergent and discriminatory validity, a high reliability and retest-reliability (Hilbert et al., 2012).

#### Body Image Avoidance Questionnaire (BIAQ)

The BIAQ is used to measure self-reported body related avoidance behavior (Rosen et al., 1991). Whereas the English original version consists of 19 items and 4 subscales, the German translation (Legenbauer et al., 2007) revealed three factors “clothing,” “social activity,” and “eating restraint” based on 11 items. Its internal consistency has been proven to be acceptable (Cronbach’s α = 0.64–0.76; present sample: Cronbach’s α = 0.85) and showed moderately stable test–retest reliability (*r*_*tt*_ = 0.64, *p* < 0.001–0.81, *p* < 0.001). To reduce codependency between body image avoidance and body checking, we decided to exclude the “eating restraint” factor (Cronbach’s α = 0.79) from our analyses, as it assesses also behavior that reflects control of food intake and not solely body related avoidance behavior.

#### Body Checking Questionnaire (BCQ)

The BCQ assesses the degree of self-reported body checking behavior and body focused control strategies (Reas et al., 2002). Its 23 items load on three separate factors: “overall appearance,” “specific body parts,” and “idiosyncratic checking.” An example item is “I check to see how my bottom looks in the mirror.” Good internal consistency (Cronbach’s α = 0.83–0.92) and test–retest-reliability have been demonstrated (*r*_*tt*_ = 0.90, *p* < 0.001; Reas et al., 2002). The German version, by contrast, reveals a single factor (Vocks et al., 2008). It also shows good internal consistency (Cronbach’s α = 0.83–0.95; present sample: Cronbach’s α = 0.96).

### Procedure

#### Diagnostic Assessment

Participants took part in exchange for €50 and were recruited from the community through newspaper announcements, our website, and from collaborating clinics. After a telephone screening, eligible participants were invited to a diagnostic session during which the EDE and SCID interviews were conducted.

#### Photo Shooting Session and Image Generation

Right after diagnostic assessment, photographs were taken and BMI determined. Participants were asked to put on a beige leotard and to stand in front of a black background. Digital pictures (termed self-picture in the following) were taken by a female experimenter from frontal, side and back view, excluding the head. The self-picture was compared to a single comparison body which was a picture taken from another participant of the same study (other-picture) that was approximately equated for BMI (±1.5 kg/m^2^). Colored body pictures were matched by eye in size and luminance^1^.

**FIGURE 1:**
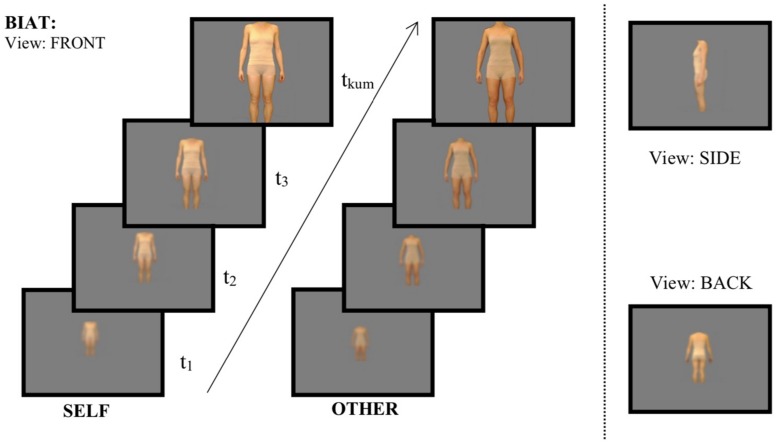
Procedure of the Body Image Approach Test (BIAT). Participants moved from low to high zoom level (increasing picture size and reducing the blurredness) by using the right mouse button. Time (t) as well as zoom level were captured for each picture. BIAT = Body Image Approach Task, FRONT = pictures presenting the front image of the participant, SIDE = pictures presenting the view on the body from the side, BACK = pictures presenting the view of the backside of the body. SELF = pictures of the own body, OTHER = pictures from bodies of weight matched control females.

Each of the three views were zoomed and blurred at the same time in 13 different levels (see Figure 1) presenting the highest blur first, ending with a fully focused high-resolution picture. Thus, the focused picture could then be zoomed closer another 11 times, so in total 24 zoom levels were possible (until maximal image extension on the 21-inch monitor).

#### Experiment

The Body Image Approach Task (BIAT) was introduced as follows: *“In the following you will be asked to evaluate pictures of yourself and of another person. But first you can look at them (front, side, back view). You can increase their size and improve the focus by pressing the right mouse button. Switch to the next slide using the middle button.”* The experimenter emphasized that participants were free to explore the pictures at their own pace, and then left the laboratory to reduce potential experimenter effects. Photos were then shown in the fixed order “self-front,” “other-front,” “self-side,” “other-side,” “self-back,” “other-back” along with the corresponding label “self” or “other” (to obviate the need for identification). The participant could advance to the next picture by clicking the middle mouse button. Image width/height was locked on all zoom levels, avoiding disproportional distortion. Viewing times and final zoom-level for each view (front, side, back view) and person (self-picture, other-picture) were registered. However, preliminary analyses indicated that viewing time did not reveal any effect that were not captured by zoom-level, too, and analyses of viewing times were therefore dropped for brevity. Final zoom-level (ranging from 1 for the initial, most remote zoom-level to 24 for maximal zoom) assessed the degree to which participants approached the pictures. In a subsequent step the same images were rated on a scale of -10 to 10 for attractiveness (“unattractive” – “attractive”) and satisfaction (“not pleased” – “pleased”). Image ratings of a subset of participants were published previously (Blechert et al., 2010). After BIAT and image ratings, several other tasks followed, assessing attentional biases regarding the images (Blechert et al., 2010). On a separate test day, several other tasks were completed by a subset of participants of the present sample (Blechert et al., 2011a, b).

### Statistical Analysis

Prior to analysis, the data was screened for potential outliers and missing data. Little’s MCAR test (Little, 1988) revealed that the data was missing completely at random (*p* > 0.999). Hence, mean imputation was used to deal with missing data. Outliers were defined as values exceeding 3.29 standard deviations above or below the mean (Tabachnick and Fidell, 2001). Accordingly, one participant had to be excluded due to unreasonably high viewing times across the different zoom-levels. Moreover, one participant had to be excluded due to technical difficulties, leaving a final sample of 62 participants. The α-criterion was set to 0.05 for all analyses. To assess differences in approach tendencies between ED and HC repeated measures analysis of variance (ANOVA) was performed. Here, a mixed model design was employed with Group (ED vs. HC) as between-subjects factor and Picture Type (self vs. other), as well as Picture Angle (front, side, back) as within-subject factors. Zoom-level served as dependent measure. In addition, two repeated measures ANOVAs with attractiveness ratings and body satisfaction as independent variables and Group (ED vs. HC) and Picture Type (self vs. other) were calculated to explore associations with other BID areas. All analyses were also performed with a 3-level group factor (AN, BN, HC). Pearson correlations assessed associations between approach behavior and specific ED behavior as well as general level of ED symptomatology for each group separately by relating total zoom-level for self- and other-pictures to the degree of BC and BA. Last, to test for the relative influence of overall ED symptomatology, BA and BC behavior as well as picture attractiveness and satisfaction ratings on BIAT zoom-level, we conducted a stepwise multiple regression. We entered ED symptom level at the first step (EDE-Q sum score), self-reported BA and BC at the second step and evaluation of the self-pictures as attractive and satisfying at the third step.

## Results

### Participant Characteristics

As indicated in Table 1, HC and ED did not differ in terms of age or BMI. However, women with ED had fewer years of education, engaged more frequently in BC, showed greater BA and higher ED psychopathology. There were no differences between AN and BN regarding these variables with the exception of BMI (*p* < 0.001). Therefore, only the results of the ANOVA with ED vs. HC as group factor are reported for reasons of brevity. Details regarding differences between AN and BN are provided as Supplementary Tables S1–S3. Furthermore, instead of full reporting, only crucial effects, mostly the interactions with Group, will be reported.

**TABLE 1:** Sample characteristics.

	**ED (*N* = 39)**		**HC (*N* = 23)**		**Statistics**		
	***M***	***SD***	***M***	***SD***	***F***	***p***	***d***
Age (in years)	24.90	6.6	26.70	4.65	3.247	0.256	−
Education (years)	11.67	1.74	12.87	0.63	66.9	< 0.01	1.01
BMI	19.19	3.66	20.29	2.21	5.368	0.199	−
BIAQ (sum)^#^	13.92	6.51	4.13	3.56	14.600	< 0.001	1.94
Social activities	4.54	3.55	0.48	1.24	44.106	< 0.001	1.70
Clothing	9.39	3.71	3.69	2.48	27.981	< 0.001	1.84
BCQ	41.76	17.34	11.51	4.68	16.584	< 0.001	2.75
EDEQ	4.01	1.26	0.44	0.55	15.084	< 0.001	3.94

*ED, eating disorder sample; HC, healthy female controls. Final zoom level corresponds to the last picture, BMI, Body Mass Index in kilogram divided by meter square; BIAQ, Body Image Avoidance Questionnaire; BCQ, Body Checking Questionnaire; EDEQ, Eating Disorder Examination Questionnaire. *d* = effect size according to Cohen’s D. #items for eating restraint not included.*

### Body Image Approach Task

#### Zoom-Level

Both groups zoomed in more toward self-pictures compared to other-pictures (*M*_*self*_ = 12.55, *SE* = 0.72; *M*_*o*__*ther*_ = 11.86, *SE* = 0.66; *F*(1,60) = 5.819, *p* = 0.019, η_*p*_^2^ = 0.088). None of the effects involving Group reached significance (all *F*s < 2.00; *p*s > 0.125). Table 2 presents mean scores and standard deviations for zoom-level separated for Picture Type and Group.

**TABLE 2:** Final zoom level scores separated by groups, picture type and angle.

	**ED (*N* = 39)**		**HC (*N* = 23)**	
	***M***	***SD***	***M***	***SD***
self-pictures total	11.92	5.37	13.19	5.45
Front	12.15	6.78	13.17	5.89
Side	11.59	5.08	13.09	6.34
Back	12.00	5.43	13.30	5.83
other-pictures total	11.56	4.74	12.15	4.99
Front	11.08	4.89	11.48	5.82
Side	12.41	5.26	12.04	5.51
Back	11.21	5.33	12.91	5.59

*Final zoom level corresponds to the last picture. self-pictures = pictures of the own body, other-pictures = pictures of another women’s body (BMI matched). ED, eating disorder sample; HC, healthy female controls; *M* = mean, *SD* = standard deviation.*

### Subjective Image Ratings

#### Attractiveness

**FIGURE 2:**
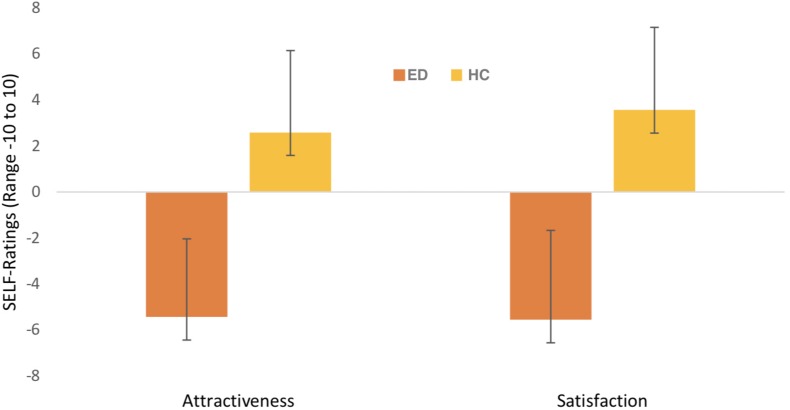
Mean scores and standard deviation for “self-picture” ratings separately for groups. Ratings refer to the dimensions attractiveness, “unattractive” – “attractive”; satisfaction, “not pleased” – “pleased”; (*x*-axis) with a range from -10 to 10 (*y*-axis). ED = eating disorder sample, HC = healthy female controls.

A significant interaction between Picture Type ∗ Group was found (*F*(1,60) = 20.003, *p* < 0.001, η_*p*_^2^ = 0.250). *Post hoc* independent *t*-tests revealed that participants with an ED rated self-pictures less attractive compared to HC (*t*(60) = −8.828, *p* < 0.001, see Figure 1), whereas no group differences were found for other-pictures (*t*(60) = −1.736; *p* = 0.088). Paired samples *t*-tests highlighted the differences in picture ratings within each group. ED patients rated self-pictures less attractive compared to other-pictures (*t*(38) = −4.900, *p* < 0.001; *M*_*o*__*ther*_ = −0.68, *SD* = 4.33). By contrast, HC’s rated self-pictures as much more attractive than other-pictures (*t*(22) = 2.124, *p* = 0.045*; M*_*o*__*ther*_ = 1.25, *SD* = 4.00). Also, a main effect for Picture Type emerged (*F*(1,60) = 4.753, *p* = 0.033, η_*p*_^2^ = 0.073) with self-pictures being overall rated more attractive than other-pictures (*M*_*self*_ = −0.995, *SE* = 0.498; *M*_*other*_ = 0.576, *SE* = 0.557). Means and standard deviations are displayed in Figure 2.

#### Satisfaction

A significant interaction emerged between Picture Type ∗ Group (*F*(1,60) = 20.001, *p* < 0.001). ED patients were less satisfied with their self-pictures compared to HC’s (*t*(60) = −9.155, *p* < 0.001). The same pattern applied for other-pictures (*t*(60) = −2.400, *p* = 0.020; *M_*ED*_* = −0.76, *SD* = 4.28; *M_*HC*_* = 1.91, *SD* = 4.16). While comparing satisfaction ratings for self- and other-pictures within one group, it became apparent that participants with EDs were much less satisfied with their self-pictures compared to pictures from others (*t*(38) = −4.703, *p* < 0.001; *M*_*s*__*elf*_ = −5.56, *SD* = 3.89; *M*_*o*__*ther*_ = −0.76, *SD* = 4.28). HC’s on the other hand, were much more satisfied with their self-picture compared to other-pictures (*t*(22) = 2.291, *p* = 0.032; *M*_*s*__*elf*_ = 3.57, *SD* = 3.60; *M*_*o*__*ther*_ = 1.91, *SD* = 4.16). In addition, a significant interaction was found for Picture Angle ∗ Group (*F*(2,120) = 3.67, *p* < 0.05). *Post hoc* independent *t*-tests demonstrated that participants with an ED were less satisfied with pictures taken from all angles compared to HC’s (all *p*’s < 0.001). Furthermore, *post hoc* paired samples *t*-tests revealed that the different photo perspectives did not influence the satisfaction ratings for participants with EDs (all *p*’s > 0.405). HCs were more satisfied when seeing front pictures compared to backside pictures (*t*(22) = −2.77, *p* < 0.05; *M*_*FrontAngle*_ = 3.46, *SD* = 3.85*; M*_*BackAngle*_ = 1.78, *SD* = 4.02).

### Associations Between BIAT Zoom-Level and ED Specific and General Symptoms

**FIGURE 3:**
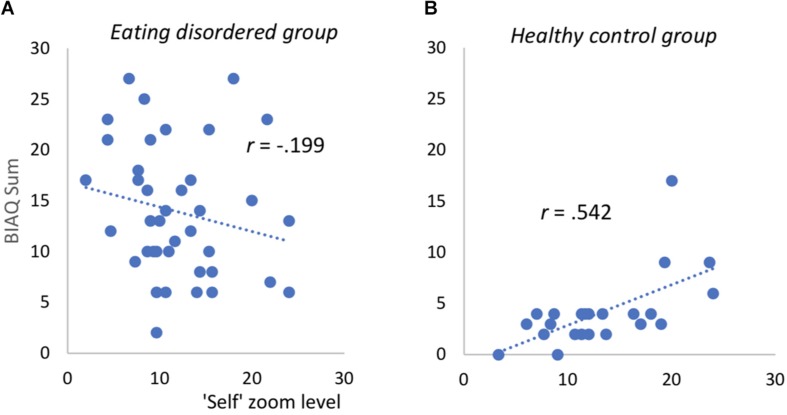
Scatterplots for association between “self”-picture zoom level and body image avoidance in eating disordered patients **(A)** and healthy controls **(B)**. Correlation remains significant after deleting one outlier on BIAQ (value of 17, healthy control group). BIAQ = Body Image Avoidance Questionnaire, computed without items for eating restraint (see text).

For patients with an ED, no significant associations between the zoom-level and BC nor the degree of BA as well as ED features and general psychopathology emerged. In HC, by contrast, BA was positively associated with the degree of zooming into self- and other-pictures (*r*(23)_*self*_ = 0.623, *p* < 0.001 see Figure 3; *r*(23)_*other*_ = 0.542, *p* = 0.008). The degree of zooming was not associated with the degree of BC (*r’s* < 0.3, *p’s* > 0.12). Also, for other-pictures, no further significant correlations emerged (see Supplementary Table S4). For self-pictures, the attractiveness ratings as well as the satisfaction ratings were significantly associated with zoom-level among HC. Also, level of ED symptomatology captured with the EDEQ total score was significantly correlated with final zoom-level among HC. For further details please see Table 3.

**TABLE 3:** Correlation coefficients for “self-pictures” in the BIAT with eating disorder and body image disturbance symptomatology among healthy controls (upper diagonal) and eating disorder patients (lower diagonal).

		**Zoom**	**Satisfaction**	**Attractiveness**	**EDEQ**	**BCQ**	**BIAQ**
Zoom	r	1	**−0.540**	**−0.704**	**0.460**	0.330	**0.623**
	p		**0.008**	**<0.001**	**0.027**	0.124	**0.001**
Satisfaction	r	0.094	1	**0.828**	**−0.489**	**−0.567**	−0.371
	p	0.567		**<0.001**	**0.018**	**0.005**	0.081
Attractiveness	r	0.041	**0.848**	1	**−0.469**	**−0.642**	**−0.469**
	p	0.802	**0.000**		**0.024**	**0.001**	**0.024**
EDEQ	r	−0.100	**−0.467**	**−0.317**	1	0.389	**0.486**
	p	0.545	**0.033**	**0.049**		0.067	**0.019**
BCQ	r	−0.041	−0.299	**−0.320**	**0.631**	1	0.224
	p	0.806	0.065	**0.047**	**<0.001**		0.304
BIAQ	r	−0.199	**−0.319**	−0.207	**0.567**	0.271	1
	p	0.226	**0.048**	0.207	**<0.001**	0.095	

*Zoom = final zoom level during the body image avoidance task (BIAT); Satisfaction = satisfaction ratings for self-pictures, Attractiveness = attractiveness ratings for self-pictures, EDEQ = Eating Disorder Examination Questionnaire total score, BCQ = Body Checking Questionnaire, BIAQ = Body Image Avoidance Questionnaire, sum score without eating restraint items; *r* = pearson correlation coefficient, *p* = probability level. Bold letters indicate statistical significance. Gray colored numbers in the lower diagonal display coefficients among ED patients, black colored numbers in the upper diagonal represents coefficients among healthy controls.*

#### Prediction of Zoom-Level

The stepwise hierarchical regression model was not significant for ED patients and therefore is only reported within the Supplementary Table S5. In contrast, the model for the HC group revealed statistically significant improvements in each step. The variables entered in the first step (EDE-Q mean score) explained 17.4% of zoom-level variance (*F*(1,21) = 5.622, *p* = 0.027) indicating a positive significant influence of ED symptomatology on final zoom-level in the BIAT. Entering self-reported levels of BA and BC increased the explained variance significantly *(p* = 0.038) and led to 35.3% of total explained variance (*F*(3,19) = 5.002, *p* = 0.010). At this step, EDE-Q sum score lost its significance and BA emerged as single significant predictor of zoom-level. BC was not of relevance. When entering the self-picture ratings in the third step (*F*(5,22) = 5.763, *p* = 0.003) explained variance increased to 52.0%. In this third step, only attractiveness ratings emerged as significant predictor. The details for this regression model are displayed in Table 4.

**TABLE 4:** Results of stepwise hierarchical regression analyses with zoom level as dependent variable (HC group).

		**B**	**SE**	**Beta**	***T***	***p***
1	(constant)	11.137	1.370		8.132	0.000
	EDEQ	4.698	1.981	0.460	2.371	0.027
2	(Constant)	7.007	2.656		2.638	0.016
	EDEQ	1.512	2.123	0.148	0.712	0.485
	BA	0.812	0.309	0.516	2.627	0.017
	BC	0.188	0.223	0.157	0.845	0.409
3	(Constant)	16.958	4.138		4.098	0.001
	EDEQ	0.933	1.894	0.091	0.845	0.409
	BA	0.510	0.286	0.324	1.786	0.092
	BC	–0.204	0.236	–0.170	–0.864	0.637
	Satis	0.202	0.421	0.130	0.480	0.637
	Attr	–1.143	0.473	–0.726	–2.413	0.027

*Zoom = final zoom level during the body image avoidance task (BIAT); EDE = Eating Disorder Examination questionnaire total score, BC = sum score of the Body Checking Questionnaire, BA = sum score without control eating items of the Body Image Avoidance Questionnaire, Satis = satisfaction ratings for SELF-pictures, Attr = attractiveness ratings for SELF-pictures, B = standardized Beta, SE = Standard error, beta = unweight Beta.*

## Discussion

The present study is the first to apply a behavioral approach task to assess BA and BC in women with a diagnosed ED in regard to images of their own body (and a matched control body). Thus, it represents an important step beyond self-report and can therefore enhance our understanding of this neglected behavioral BID component. The results can be summarized as follows: First, our picture ratings on attractiveness and satisfaction replicated previous findings of a strongly negative cognitive affective BID in both AN and BN – both ED groups rated their self-picture much more negative as the BMI-matched other picture, with the reverse being true in healthy controls. Despite these robust group differences, the same patients did not approach their self-pictures differentially in the picture zooming procedure of the BIAT. Furthermore, the questionnaire measures of BID, the BCQ and the BIAQ did not correlate with the new BIAT in the ED sample. Only in HCs did the BIAT correlate strongly and positively with self-reported BA, suggesting initial evidence for construct validity with regard to the lower levels of body image avoidance scores of healthy controls. Considering associations between BA, ED symptom level and body satisfaction, the regression analyses within the healthy control group revealed that ratings of perceived attractiveness of the own body was the most relevant predictor of approach behavior as measured in the BIAT. This underlines the critical role of body related attitudes also for behavioral features in non-eating disordered women.

These results raise several questions: First, the BIAT did not differentiate between ED and HC and as such did not complement findings from studies using AAT to capture approach-avoidance behavior as a behavioral assessment. Given that the BIAT seems a valid tool in assessing disorder specific psychopathology in various psychiatric conditions, in particular anxiety (Deacon and Olatunji, 2007; Olatunji et al., 2007; Brady and Lohr, 2014) the question as to why ED patients do not respond to their own, disliked self-images in a similar way as phobic patients to disorder specific stimuli (e.g., spiders) is puzzling. It is possible that the focus on approach behavior – namely the start with the most blurred and small picture size going to the highest solution and biggest size – is not as sensitive in highly pathological samples as the AAT design which allows participants to “move” pictures away or toward oneself and which focusses on reaction times. On the other hand, the only study that applied an AAT in (questionnaire-defined) patients with eating disturbances and BID (Leins et al., 2018) did not include self-images and it is therefore not easy to compare the two studies. Interestingly, Leins et al. (2018) also failed to replicate an approach bias toward thin pictures, illustrating how difficult it generally is to reliably capture approach-avoidance behavior toward body images. This might also be caused by their use of computer-generated bodily stimuli (avatars; e.g., Woud et al., 2011; Leins et al., 2018; Dondzilo et al., 2019). We tried to overcome this limitation by using self-pictures compared to other-pictures, however, this did not lead to a more differential pattern between groups. It is possible that pictures of one’s own body without head displayed on a computer screen did not elicit strong enough automatic approach or avoidance or body-related anxiety. However, looking at the ratings for self- and other-pictures, it is obvious that the negative attitude concerning the own body had been elicited in ED patients, as these reported significantly more negative attitudes toward their own body compared to the bodies of other females. Also, EDs showed significantly stronger negative attitudes compared to HCs. This is in line with former research showing implicit negative body-related attitudes in women with AN and BN compared to healthy women (e.g., Voges et al., 2018).

A further point that is puzzling is that self-report assessments neither for BA nor for BC in ED patients correlated with the results in the BIAT. Former research indicates that BC and BA serve different functions implying alternated representations depending on situational contexts (e.g., Shafran et al., 2004; Bailey and Waller, 2017; Radix et al., 2018). BC includes primarily repetitive actions to check one’s shape and weight (e.g., weighing, pinching in the skin, examining body parts in the mirror or seeking reassurance from others), whereas BA comprises actions that hinder the confrontation with one’s shape and weight. Recent evidence emphasizes the role of social context for BA and BC by pointing out that social anxieties and fear of negative evaluation in patients with various EDs serve as a potential mediator between ED symptomatology and BC and BA behavior (Radix et al., 2018). As such, it might be that the lack of social context and the rather “artificial” pictures without head presented during the BIAT rather triggered BA instead of BC. The impact of social context and social comparison might also account for a lack of associations between BA and BIAT zoom-levels in the ED sample; it might be that AN and BN patients are more used to look at their own bodies and that social context and fear of evaluation is necessary to trigger compensatory safety behaviors (e.g., Utschig et al., 2010), whereas in healthy female controls looking at pictures of the own body is not that usual and therefore triggers body dissatisfaction which is associated with BA. Thus, the approach behavior as assessed with the BIAT might tap into a different function that is not presented in highly pathological avoiders and thus does not detect differential effects in this group.

Interestingly, in the HC group, higher avoidance in the BIAQ (our measure of BA) went along with higher zoom-levels for self- as well as other-pictures in the BIAT. This dovetails with the perplexing finding that some individuals report *both* body-related avoidance behavior on the BIAQ *and* frequent body checking (as evident from a positive correlation of both instruments in other samples (e.g., Campana et al., 2013; Legenbauer et al., 2017; Radix et al., 2018; and the present sample). We verified that control females were healthy via a structured clinical interview, nevertheless there seemed to be some behavioral body related avoidance or checking. Hence, on subclinical levels, one could think of this pattern as some kind of ambivalent situation, being dissatisfied and still vigilant to it. Thus, it seems that there were at least some individuals in the HC group reporting some BA behavior and this is expressed in lower BIAT zoom-levels. An even better measure of BA, however, seems to be the image attractiveness ratings: when taking these into account, BIAQ scores no longer predict zoom-level. This is in contrast to former results that emphasized the influence of body dissatisfaction and drive for thinness. In the present analyses, self-rated attractiveness was the most powerful predictor pointing toward a dominant role of the thin ideal and body related attitudes on approach behavior. Theories of social comparison point out that most information is gained from comparing against similar others, so this might have taken place here (Goethals and Darley, 1977).

Besides the strength of a clinical sample diagnosed with a clinical structured interview and the thoroughly performed assessment, there are some limitations that have to be mentioned. *First*, the present study includes various ED categories which might have conflated differential effects for single ED categories. We controlled for such differential effects between AN and BN patients, but failed to find any in relation to BA, BC or BIAT or sample characteristics. It may be that the ED subsamples were too small to detect smaller effects. Small sample size also affects the correlational analysis within the HC group and calls for replication in a larger sample. *Second*, it might also be that methodological issues of the BA self-report tool account for the lack of associations in ED. We used the BIAQ that has been developed in student samples and whose original structure has not been replicated in several studies (Legenbauer et al., 2007; Campana et al., 2013; Brytek-Matera and Rogoza, 2016). It has to be considered that within the German version, the original factor structure had not been replicated and that items in relation to eating loaded on a factor that reflects rather control than avoidance (Legenbauer et al., 2007). To minimize possible dependencies between BA and BC self-report, we did not include the eating control items in the sum score. Thus, the items of the BIAQ used in the analyses relate to clothing and social activities. These are behaviors that might reflect other components of avoidance behavior than those captured within the BIAT. Also, it is possible that the self-report assessments do not adequately capture the behavioral symptoms such as body checking and body image avoidance behaviors, thus veiling any relationship with the BIAT. *Third*, and related to the validity of the BIAT task, avoidance behavior might not be well captured, because the BIAT does not explicitly include the possibility to push the picture away (as possible in a classical joystick-AAT). Yet, participants were free to use the middle mouse button to advance immediately to the next photo. Similarly, we cannot be sure that zooming in and increasing focus in the BIAT corresponds to naturalistic body related checking behaviors even though it corresponds well to the classical bodily inspection in a mirror. *Fourth*, while we chose one single “other” body per participant and made sure that it matches the participant’s BMI ±1.5 kg/m^2^) we did not use a different “other” body for each participant. Thus, variance in the “other” category might be restricted. *Finally*, by displaying the whole body, differences regarding different body parts could not be taken into account which awaits future study.

## Conclusion

The present study represents a first step toward an assessment tool for the behavioral component of body image. So far, it seems that what is measured by the current BA questionnaire in healthy individuals goes along with more detailed checking and close examination of body related pictures of ones’ own body, maybe in search for signs of failures or deviations and a similar examination of relevant comparison bodies (other’s bodies). At higher levels of BA – as here in the ED patient group – this relationship is lost or countered by additional processes that we could not measure here. In other words, the BIAT does not seem to capture the body image satisfaction or the behavioral components of BID in ED patients or it conflates counteracting influences such as BA and BC. Further experimentation is needed to adapt measures of behavioral body image components to the processes engaged in patients with ED during confrontation with body images. Inclusion of social context information and mood (anxiety) or stress induction may be a next step to enhance the understanding of the behavioral BID component in EDs.

## Data Availability Statement

The datasets generated for this study are available on request to the corresponding author.

## Ethics Statement

The study was approved by the ethics committee of the German Society for Psychology. The study protocol was conducted in accordance with the Declaration of Helsinki (revised 1983). Written informed consent was provided by all participants, who were aware that they could withdraw from the experiment at any time without further consequences.

## Author Contributions

JB designed the study, collected the data, and participated in the analyses and manuscript writing. EN, AR, and TL analyzed the data. TL and AR conducted the literature review and wrote the first draft of the manuscript. All authors approved the final version of the manuscript.

## Conflict of Interest

The authors declare that the research was conducted in the absence of any commercial or financial relationships that could be construed as a potential conflict of interest.

## Footnotes

1 Images were not compared on any algorithmically derived index as it is not clear which parameter (e.g., edge detection, spectral complexity, contrast, brightness, and color composition) would be relevant. However, to prevent any gross differences, the last author individually edited the photos to make them as similar as possible, e.g., in color saturation or size.
