# Supplementary material for: The Body Image Approach Test (BIAT): A Potential Measure of the Behavioral Components of Body Image Disturbance in Anorexia and Bulimia Nervosa?
Source: Front Psychol. 2020 Jan 31;11:30. doi: 10.3389/fpsyg.2020.00030 (PMC7005054; doi:10.3389/fpsyg.2020.00030)
Supplement: Supplementary file 1 [file Table_1.docx]

Table S1: Differences between AN and BN regarding sample characteristics

|  |  | |  |  |
| --- | --- | --- | --- | --- |
|  | AN (N=21) | | BN (N=18) | |
|  | *M* | *SD* | *M* | *SD* |
| Age (in years) | 23.19 | 4.55 | 26.89 | 8.08 |
| Education (years) | 11.71 | 1.65 | 11.61 | 1.88 |
| BMI | 16.58 | 1.30 | 22.24 | 3.11 |
| BIAQ (sum)^#^ | 15.14 | 6.35 | 12.5 | 6.57 |
| Social activities | 5.52 | 3.60 | 3.39 | 3.2 |
| Clothing | 9.62 | 3.57 | 9.11 | 3.95 |
| BCQ | 43.28 | 18.98 | 39.98 | 15.57 |
| EDEQ | 3.89 | 1.44 | 4.15 | 1.03 |

Note: AN = patients with a diagnosed Anorexia nervosa, BN = patients with a diagnosed Bulimia nervosa. BMI = Body Mass Index in kilogram divided by meter square, BIAQ = Body Image Avoidance Questionnaire, BCQ = Body Checking Questionnaire, EDEQ = Eating Disorder Examination Questionnaire
