# Supplementary material for: The Body Image Approach Test (BIAT): A Potential Measure of the Behavioral Components of Body Image Disturbance in Anorexia and Bulimia Nervosa?
Source: Front Psychol. 2020 Jan 31;11:30. doi: 10.3389/fpsyg.2020.00030 (PMC7005054; doi:10.3389/fpsyg.2020.00030)
Supplement: Supplementary file 2 [file Table_2.docx]

Table S2: Mean and standard deviation for AN and BN

|  | AN (N=21) | | BN (N=18) | |
| --- | --- | --- | --- | --- |
|  | *M* | *SD* | *M* | *SD* |
| self-pictures |  |  |  |  |
| front | 12.76 | 7.36 | 11.44 | 6.17 |
| side | 11.67 | 5.43 | 11.5 | 4.79 |
| back | 12.71 | 6.21 | 11.5 | 4.79 |
| other-pictures |  |  |  |  |
| front | 11.48 | 5.88 | 10.61 | 3.52 |
| side | 12.48 | 5.38 | 12.33 | 5.27 |
| back | 11.24 | 5.75 | 11.17 | 4.95 |

Note: AN = patients with diagnosed Anorexia nervosa, BN = patients with diagnosed Bulimia nervosa, self-pictures = zoom levels for pictures that displayed one’s own body, other-pictures = zoom level for pictures that displayed other women’s bodies. M = Mean, SD = Standard deviation.
