# Supplementary material for: The Body Image Approach Test (BIAT): A Potential Measure of the Behavioral Components of Body Image Disturbance in Anorexia and Bulimia Nervosa?
Source: Front Psychol. 2020 Jan 31;11:30. doi: 10.3389/fpsyg.2020.00030 (PMC7005054; doi:10.3389/fpsyg.2020.00030)
Supplement: Supplementary file 3 [file Table_3.DOCX]

Table S3: Differences in self-ratings for self- and other-pictures separated for AN and BN

|  | BN | AN |
| --- | --- | --- |
| self-rated  satisfaction (m, sd) | -6.10  (3.61) | -5.10 (4.12) |
| attractiveness (m, sd) | -5.76  (3.33) | -5.16  (3.52) |
| other -rated  satisfaction (m, sd) | 0.15  (4.39) | 1.54  (4.13) |
| attractiveness (m, sd) | -.04  (4.72) | -1.22 (4.00) |

Note: BN = patients with diagnosed Bulimia nervosa, AN = patients with diagnosed Anorexia nervosa, m = mean, sd = standard deviation, satisfaction = body satisfaction ratings for pictures of the one’s own or other women’s bodies, attractiveness ratings for pictures with one’s own or other women’s bodies.
