# Supplementary material for: The Body Image Approach Test (BIAT): A Potential Measure of the Behavioral Components of Body Image Disturbance in Anorexia and Bulimia Nervosa?
Source: Front Psychol. 2020 Jan 31;11:30. doi: 10.3389/fpsyg.2020.00030 (PMC7005054; doi:10.3389/fpsyg.2020.00030)
Supplement: Supplementary file 4 [file Table_4.docx]

Table S4: Correlation coefficients for ‘other-pictures’ in relation to BIAT zoom level, body avoidance, body checking and body image disordered attitudes as well as eating disorder symptoms

|  |  |  |  |  |  |  |  |
| --- | --- | --- | --- | --- | --- | --- | --- |
|  |  | zoom | BIAQ | BCQ | satisfaction | attractive | EDEQ |
| zoom | *r* | 1 | -.133 | -.01 | -.082 | -.156 | -.064 |
|  | *p* |  | .418 | .996 | .619 | .342 | .699 |
| BIAQ | *r* | -.133 | 1 | .271 | -.178 | -.304 | .567^**^ |
|  | *p* | .418 |  | .095 | .278 | .060 | .000 |
| BCQ | *r* | -.001 | .271 | 1 | -.080 | -.001 | .631^**^ |
|  | *p* | .996 | .095 |  | .629 | .993 | .000 |
| satisfaction | *r* | -.082 | -.178 | -.080 | 1 | .866^*^ | -.117 |
|  | *p* | .619 | .278 | .629 |  | .000 | .477 |
| attractive | *r* | -.156 | -.304 | -.001 | .866^**^ | 1 | -.138 |
|  | *p* | .342 | .060 | .993 | .000 |  | .401 |
| EDEQ | *r* | -.064 | .567^**^ | .631^**^ | -.117 | -.138 | 1 |
|  | *p* | .699 | .000 | .000 | .477 | .401 |  |

Note: BIAQ = Body Image Avoidance Questionnaire, BCQ = Body Checking Questionnaire, satisfaction = rated body satisfaction for ‘other-pictures’, attractive = attractiveness ratings for ‘other-pictures’, EDEQ = Eating Disorder Examination Questionnaire
