# Supplementary material for: The Body Image Approach Test (BIAT): A Potential Measure of the Behavioral Components of Body Image Disturbance in Anorexia and Bulimia Nervosa?
Source: Front Psychol. 2020 Jan 31;11:30. doi: 10.3389/fpsyg.2020.00030 (PMC7005054; doi:10.3389/fpsyg.2020.00030)
Supplement: Supplementary file 5 [file Table_5.docx]

Table S5: Results of stepwise hierachical regression analyses among ED patients

|  | Regr. coeff. B | SE | beta | T | *p* |
| --- | --- | --- | --- | --- | --- |
| (constant) |  |  |  |  |  |
| EDEQ | -.426 | .697 | -.100 | -.611 | .545 |
| (constant) |  |  |  |  |  |
| EDEQ | .057 | 1.073 | .013 | .054 | .958 |
| BIAQ | -.172 | .167 | -.208 | -1.026 | .312 |
| BCQ | .002 | .067 | .007 | .034 | .973 |
| (constant) |  |  |  |  |  |
| EDEQ | .550 | 1.166 | .129 | .472 | .640 |
| BIAQ | -.166 | .168 | -.201 | -.985 | .332 |
| BCQ | -.024 | .070 | -.077 | -.342 | .735 |
| attractiveness | -.699 | .525 | -.443 | -1.333 | .192 |
| satisfaction | .611 | .485 | .443 | 1.259 | .217 |

Note: ED = Eating disorder, EDEQ = Eating Disorder Examination Questionnaire, BIAQ = Body Image Avoidance Questionnaire, BCQ = Body Checking Questionnaire, attractiveness = attractiveness = attractiveness ratings for pictures that displayed one’s own body. T = t-test, Regr. Coeff. B = Regression Coefficient B. SE = standard error.
